# Effect of the Length of Oat Hay on Growth Performance, Health Status, Behavior Parameters and Rumen Fermentation of Holstein Female Calves

**DOI:** 10.3390/metabo11120890

**Published:** 2021-12-20

**Authors:** Jianxin Xiao, Tianyu Chen, Gibson Maswayi Alugongo, Muhammad Zahoor Khan, Tingting Li, Jing Ma, Shuai Liu, Wei Wang, Yajing Wang, Shengli Li, Zhijun Cao

**Affiliations:** Beijing Engineering Technology Research Center of Raw Milk Quality and Safety Control, State Key Laboratory of Animal Nutrition, College of Animal Science and Technology, China Agricultural University, Beijing 100193, China; xiaojianxin-dairy@cau.edu.cn (J.X.); chentianyu@cau.edu.cn (T.C.); lb20163040001@cau.edu.cn (G.M.A.); zahoorcau@cau.edu.cn (M.Z.K.); litingting@newhope.cn (T.L.); s20193040572@cau.edu.cn (J.M.); liushuaicau@cau.edu.cn (S.L.); wei.wang@cau.edu.cn (W.W.); yajingwang@cau.edu.cn (Y.W.); lishengli@cau.edu.cn (S.L.)

**Keywords:** calf, oat hay length, growth, rumen fermentation, health, behavior

## Abstract

The aim of this study was to evaluate the effect of the length of oat hay on the performance, health, behavior, and rumen fermentation of dairy calves. For this purpose, two hundred and ten healthy two-day-old Holstein dairy calves were randomly allocated into three groups: basic diet (calf starter) without hay (CON), or a basic diet with oat hay at either long (OL: 10–12 cm) or short (OS: 3–5 cm) length cut. The basic diet was fed from day 4, while the hay was offered from day 14. All calves were weaned at day 56 and remained in their individual hutches till the end of the trial (day 70). Calf starter intake and fecal scores were recorded daily. Bodyweight, body size, and rumen fluid samples were collected biweekly before weaning and weekly after weaning. Overall, providing oat hay (OS and OL) in the diet increased the body weight, starter intake, and average daily gain compared to the CON group. Similarly, feeding oat hay improved rumen fermentation. More specifically, hay enhanced the rumen pH and changed the rumen fermentation type. Hay fed calves spent more time on rumination but less time performing abnormal behaviors compared to control. As it can be concluded, feeding oat hay to calves enhances the growth performance, rumen fermentation, and normal calf behaviors, implying improved animal welfare irrespective of the hay length.

## 1. Introduction

Improved morphological and metabolic functions of the rumen are important at early stage of calves’ life. The normal development of the rumen epithelium depends on ingesting solid feed, and especially concentrates that produce butyric and propionic acid [1]. In the first few weeks of life, the calf cannot consume sufficient solid feed and depends mainly on milk for its maintenance, growth, and development needs. However, milk bypasses the rumen via the esophageal groove into the abomasum and small intestine, where it is digested and absorbed [2] and may restrain the rumen development in calves [3]. Although solid feed intake increases with age, it is not until complete weaning from milk that the calf can consume adequate solid feed to support its nutritional needs, providing required substrates for growth and development of the rumen mass papillae [4].

Feeding diets with a high percentage of concentrates can predispose calves to low rumen pH due to rapid ferment of easily fermentable carbohydrates, leading to the accumulation of rumen fermentation products [5]. On the contrary, the large particle size and rough surfaces of the forage can stimulate rumination and increase the flow of saliva into the rumen [6], which can alleviate the low rumen pH. Moreover, due to the small particle size of concentrates, plaque formation on the rumen epithelium increases, eventually leading to ruminal papillae hyperkeratosis and rumen mucosa thickening [7,8,9] and ultimately a decrease in VFA absorption [10].

The physical form of the forage is a key factor that influences nutrient digestion and growth performance in calves. Forage can be cut to different lengths before feeding calves, resulting in different forms and texture. Compared to ground grass hay, calves that were fed chopped grass hay had improved total DMI, and nutrient digestibility and displayed reduced rates of non-nutritive behavior [11]. Another research project reported that calves had higher ADG when a diet containing 25% long alfalfa hay and calf starter was provided [12]. Norouzian et al. [9] reported that long alfalfa hay was more advantageous than short alfalfa hay (fine: 2 mm, long: 3 to 4 cm, as geometric mean) in promoting the development of rumen (as the former stays longer). Mirzaei et al. [8] reported that feed intake and weaning body weight were improved as the physical size of alfalfa hay increased up to 8% of the basic diet. However, no differences were observed in calves fed at 28% alfalfa of the basic diet in the same trial. Indeed, some studies have completely discouraged feeding calves long hay [13,14] as they associate hay feeding with low starter intake (partly due to rumen fill and limitation in forage digestion) and thus decreased energy intake [15,16]. On the other hand, although some farms use oat hay to feed calves, there was little literature on the physical form of oat hay supply (mostly on alfalfa hay). Castells et al. [17] compared the effects of different forage sources (oat hay, barley straw, triticale silage, and alfalfa hay) on calves and found higher DMI and ADG in the oat hay group compared with the alfalfa hay group. Many factors affect calf behavior, including environment, weaning, and feeding management [18]. Moreover, the forage sources [17], physical form [11], supplementation level and particle size [8] could also influence calf behavior. Understanding calf behavior can help farmers better manage their young stock [19]. Phillips [20] found that providing hay to calves could reduce the behavior of bedding intake and the licking of the bucket and pen. Montoro et al. [11] also found that compared with finely ground (2 mm) grass hay, calves spent less time on non-nutritive oral behaviors when calves receiving coarsely chopped (3–4 cm) grass hay.

Despite extensive research on dairy calves, controversy remains on whether calves should be fed hay in the pre-weaning period. Moreover, dairy farmers have no clear recommendations on the optimal length of hay fed to dairy calves or may lack the capacity to cut hay finely. We hypothesized that the length of oat hay cut but not feeding hay itself may affect calf performance. Thus, the objective of this study was to determine the effects of feeding long and short oat hay on calf growth performance, rumen fermentation, health status, and calf behavior.

## 2. Results

### 2.1. BW, ADG and Starter Intake

**Figure 1:**
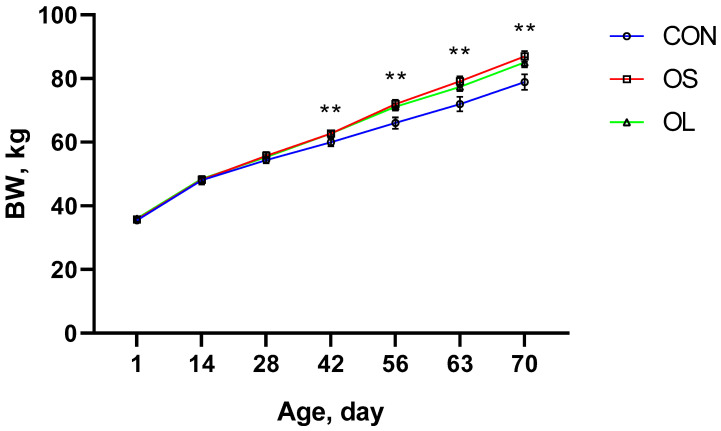
BW for Holstein’s calves fed a basic diet without hay (CON: empty circles; red line) and inclusion of short oat hay (OS: 3–5 cm, empty squares; green line) or inclusion of long oat hay (OL: 10–12 cm, empty triangles; blue line). Differences between CON and hay treated groups are represented by an asterisk (*p* < 0.05, denoted by **).

**Figure 2:**
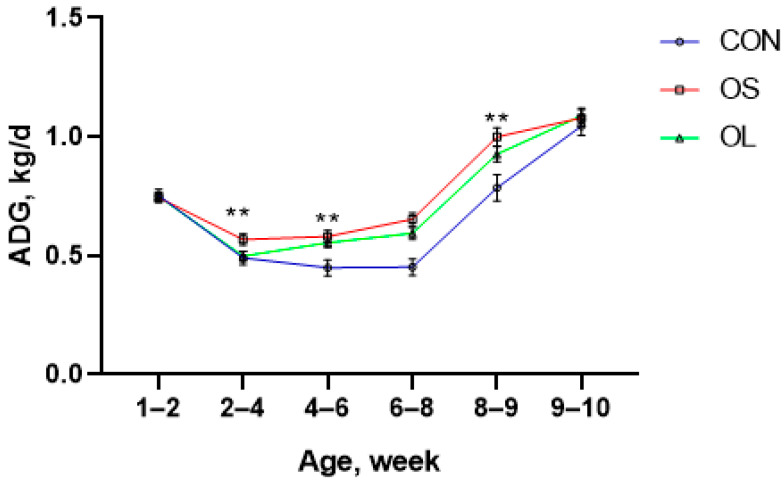
Mean ADG for Holstein female calves fed a basic diet without hay (CON: empty circles; red line) and inclusion of short oat hay (OS: 3–5 cm, empty squares; green line) or inclusion of long oat hay (OL: 10–12 cm, empty triangles; blue line). Differences between CON with hay are represented by an asterisk (*p* < 0.05, denoted by **).

**Figure 3:**
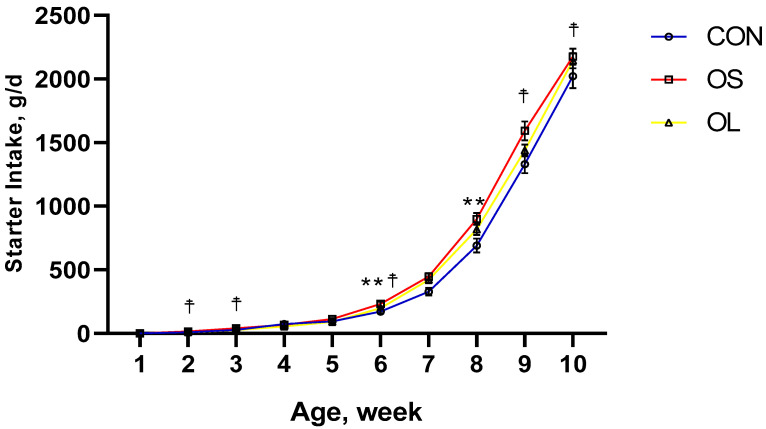
Calf starter intake for Holstein female calves fed a basic diet without hay (CON: empty circles; red line) and inclusion of short oat hay (OS: 3–5 cm, empty squares; green line) or inclusion of long oat hay (OL: 10–12 cm, empty triangles; blue line). Differences between CON with hay are represented by an asterisk (*p* < 0.05, denoted by **). Differences between OS with OL are represented by a cross (*p* < 0.05, denoted by ☨).

BW, ADG, and starter intake data are shown in Table 1 and Figure 1, Figure 2 and Figure 3; BW was affected by the addition of hay to the diet, since higher BW was observed in calves fed hay (*p* < 0.01) during the whole trial period (Figure 1). There were no differences observed in BW from day 1 (week 1) to 28 (week 4). Nevertheless, calves fed hay had a higher BW than the CON (*p* < 0.01) group from the period ending on day 42 (week 5–6) until the end of the study on day 70 (week 10). At the same time, calves fed short oat hay had higher ADG than CON (*p* < 0.01) and long oat hay treatment (*p* < 0.01) during pre-weaning and the entire trial (Table 1). However, no significant differences between short oat hay and long oat hay treatments were reported in post-weaning calves. Consistently, calves fed with hay had higher ADG between weeks 2–4 and weeks 8–9 (*p* < 0.05) except for weeks 6–8 (Figure 2). The starter was greater in hay-fed groups compared to CON during pre-weaning (*p* < 0.05) and entire trial (*p* < 0.05) periods. In addition, short oat hay calves consumed more calf starter than the long oat hay calves in week 2 to 3 (*p* < 0.05), week 6 (*p* < 0.05) and week 9 to 10 (*p* < 0.05).

### 2.2. Body Growth Parameters

Data of body structural measurements (height, body length, heart girth, abdominal girth, circumference of cannon bone) are presented in Table 2. Hay treatments did not influence body height and circumference of cannon bone during the three-time periods (pre-weaning, days 1–56; post-weaning, days 57–70 and entire trial, days 1–70). No differences were observed for body length and heart girth during the pre-weaning period. However, body length (*p* < 0.01) and heart girth (*p* < 0.05) in calves fed hay were greater during post-weaning and the entire trial compared to CON. The hay groups had greater abdominal girth (*p* < 0.01) than the CON group during the three time periods. In the aforementioned results, there was no difference between short oat hay and long oat hay groups.

### 2.3. pH and NH_3_-N

**Figure 4:**
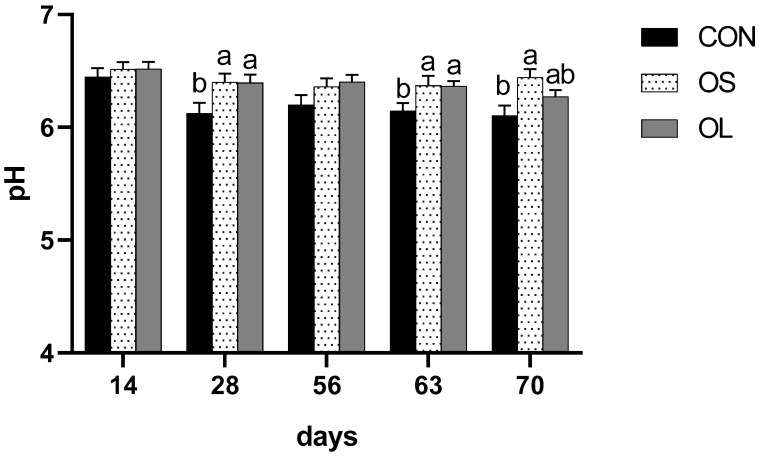
Ruminal pH for Holstein female calves fed basis diet without hay (CON: black bar) and inclusion of short oat hay (OS: 3–5 cm, spots bar) or inclusion of long oat hay (OL: 10–12 cm, gray bar). Different letters within a time point indicate significant differences among treatments (*p* < 0.05). Different lowercase letters (a, b, ab) above the bar indicate significant difference (*p* *<* 0.05).

**Figure 5:**
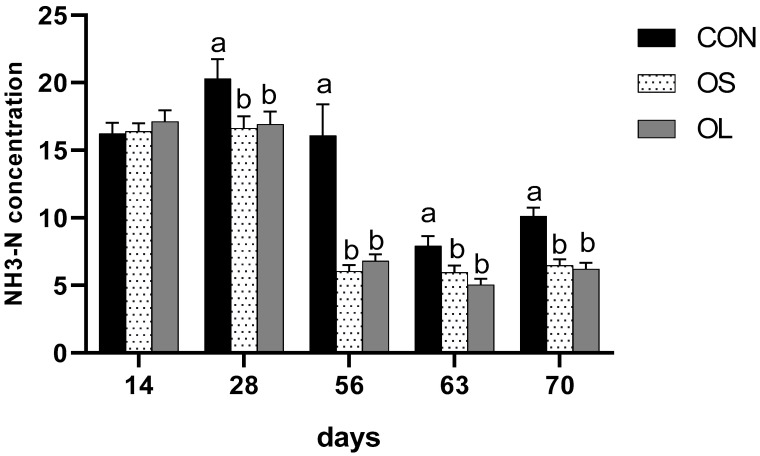
NH_3_-N concentration for Holstein female calves fed basis diet without hay (CON: black bar) and inclusion of short oat hay (OS: 3–5 cm, spots bar) or inclusion of long oat hay (OL: 10–12 cm, gray bar). Different letters within a time point indicate significant differences among treatments (*p* < 0.05). Different lowercase letters (a, b) above the bar indicate significant difference (*p* *<* 0.05).

A significant difference was observed in rumen pH, since hay-fed calves had higher values than CON during pre-weaning (*p* < 0.05), post-weaning (*p* < 0.01) and entire trial (*p* < 0.01) periods (Table 3). However, no differences were observed between the OS and OL groups. In different weeks (Figure 4), calves fed hay had higher rumen pH than CON on day 28 (week 4, *p* < 0.05), 63 (week 9, *p* < 0.05), 70 (week 10, *p* < 0.05). The concentration of rumen ammonia nitrogen was affected by hay feeding; calves fed hay had decreased NH3-N concentration compared to CON calves during pre-weaning (*p* < 0.01), post-weaning (*p* < 0.01) as well as an entire trial (*p* < 0.01). In different weeks (Figure 5), CON group had higher NH3-N concentration on day 28 (week 4, *p* < 0.05), 56 (week 8, *p* < 0.01), 63 (week 9, *p* < 0.05), 70 (week 10, *p* < 0.01) compared with OS and OL.

### 2.4. Rumen Volatile Fatty Acids

Acetate concentration was not different among groups during pre-weaning and entire trial periods (Table 4). A higher proportion of acetate was observed in OS (*p* < 0.05) and OL (*p* < 0.05) groups during the post-weaning period compared to CON. The CON group had a significantly higher propionate proportion than hay (OL and OS) (*p* < 0.05) treatments during post-weaning, while OS group showed a lower propionate proportion than CON and OL during the entire trial. Calves receiving long oat hay had a lower butyrate proportion than the short oat hay (*p* < 0.01) during the pre-weaning and post-weaning periods. The CON group had higher TVFA than OL (*p* < 0.05) during the pre-weaning and entire trial period. Calves fed with hay had higher value of C2/C3 than CON in post-weaning (*p* < 0.05) period. However, no differences were observed between short and long oat hay groups.

### 2.5. Calf Health

The effect of oat hay length on calf health (Table 5) showed that different treatments had no significant influence on the diarrhea frequency. The diarrhea frequency changed with time and was higher in pre-weaning compared to the post-weaning period (*p* < 0.01). No differences were observed on diarrhea duration among the treatments at different periods. No significant change in the occurrence of pneumonia among different groups was found during the pre-weaning period.

### 2.6. Calf Behavior

Time spent on each behavior at day 57, 63, and 70 is presented in Table 6. The OS and OL calves spent less time standing (*p* < 0.05) and OS spent more time lying (*p* < 0.05) compared with CON during these three days. The CON group calves spent more time on eating starter than OS and OL groups (*p* < 0.05). However, the length of hay did not affect the time the calf spent eating hay. No difference was found among treatments in drinking behavior. The calves supplemented with hay (*p* < 0.01) in the diet spent more time than CON ruminating. Furthermore, our findings showed that calves receiving hay (*p* < 0.01) shown a reduction in the time spent on abnormal behavior and head out of the pen.

## 3. Discussion

### 3.1. Calf Growth Performance

Providing hay, especially long particle-sized hay, increases salivary secretion and buffers rumen fluid in dairy calves [5,6,21]. Furthermore, it improves the rumen environment, increases dry matter intake, and contributes to greater BW [11,22,23]. The reason why feeding hay resulted in heavier calves may be the cause of increasing muscularis mucosa, weight and volume of the rumen and subsequently could enhance the feed intake and BW [8,10,24,25,26]. To some extent, early in life, the rumen is not fully developed and is less likely to digest hay efficiently. Thus, longer as compared to shorter hay particles may stay longer in the rumen due to difficulties in digestion and low passage rate in the gut [16] which may cause an increase in BW due to greater gut fill [11]. Our results showed no difference in BW between OL and OS fed calves although both were heavier than CON from day 42 to the end of the study at day 70, suggesting that calf BW could be positively influenced by hay feeding, irrespective of its cut length. Although there was no difference in BW between OS and OL, ADG was higher in the OS. In our study we fed OS and OL ranging from 3 cm to 5 cm and 10 cm to 12 cm, respectively. In a previous study, the length of longer and short grass hay was 3 cm to 4 cm (CRS) and 2 mm (FN) [11], respectively, and found that calves fed CRS had greater performance (higher feed intake and nutrient digestibility). Therefore, hay for calves needs a moderate length, neither too long nor too short, 3–5 cm may be the best choice. Castells et al. [10] reported positive outcomes in ADG when calves were fed chopped oat hay in the pre-weaning period. The importance of hay in young ruminants was demonstrated more than six decades ago. As early as 1962, Tamate et al. [25] demonstrated that hay supplementation was important in stimulating muscularis and promoting rapid growth of the rumen. A well-developed rumen enhances the production and absorption of VFAs and increases the output of microbial protein, which can be utilized for growth [27]. Thus, the low ADG in CON calves was most likely linked to decreased availability of nutrients to support growth. On the other hand, compared with OS, the OL might have filled more space in the rumen and thus reduced the overall solid feed intake [16]. Consequently, a decrease was reflected in the ADG of OL calves during the entire trial compared with OS in our study. Calves fed short-cut hay consumed more calf starter. Similarly, Montoro et al. [11] reported that providing chopped grass hay (3–4 cm) to young calves could improve feed intake by improving gut fill. An increase in gut fill might accommodate and digest more solid feed [28]. In addition, the increased availability of feed substrates in the rumen can enhance the production of VFA, which in turn stimulates rumen epithelial development [7].

The measurements of the structural (body height, body length, heart girth, abdominal girth, circumference of cannon bone) are good indicators of the calf’s growth, feeding, and management conditions. The effect of hay feeding on structural measurements has shown inconsistencies in many studies. Khan et al. [24] had reported that the structural measurements were not improved in calves fed grass hay (1.2 ± 0.4 cm) compared to texturized starter. Similarly, Hosseini et al. [29] reported no differences in hip height, body length, body barrel, heart girth and wither height between starter feed and starter feed plus 15% chopped alfalfa hay (3 mm). However, Gasiorek et al. [30] reported higher hip height on OH (starter feed containing 10% DM basis chopped oat hay) compared with starter feed only. In the present study, calves fed with hay had higher body length, heart girth, and abdominal girth during the entire trial period, mostly due to greater growth in the post-weaning period.

### 3.2. Rumen Fermentation

In agreement with previous studies, our study showed that feeding hay increases ruminal pH in calves [7,8,31] compared to the CON calves. This observation was independent of the hay cut length. Calves might take a longer time ruminating due to more frequent regurgitation of the long particles that are occupying greater volume in the rumen. Consequently, more saliva, which buffers the acidic rumen pH is produced [6]. Suarez-Mena et al. [32] investigated and found no effect of straw of different particle sizes (0.82, 3.04, 7.10, and 12.7 mm as geometric mean) mixed at the rate of 5% in the calf starter on rumen fermentation and pH in pre-weaning dairy calves. Similarly, Mirzaei et al. [8] compared two different particle sizes (medium, 2.92 mm; or long, 5.04 mm as geometrical means) of alfalfa hay supplemented at different levels (low, 8%; or high, 16% on DM basis) in calf starter and found no effect on VFA production and rumen pH in calves. Terre et al. [33] by adding chopped oat hay (49.2% between 8 and 20 mm) in the pelleted starter and found it could improve the ruminating behavior and result in a higher pH compared with starter only. Forage length which could partially influence the time spent on rumination was an important physical form in maintaining optimal rumen pH [34]. Mirzaei et al. [8] reported that in calves provided with forage of different sizes (alfalfa hay: short = 1.96 mm or long = 3.93 mm; and wheat straw: short = 2.03 mm or long = 4.10 mm as geometric mean), rumination time increased in those that received forage with long particle sizes. In our experiment, no differences were observed on the rumen pH between OS and OL treatments, probably due to a lack of controlling the level of forage supplementation. Calf performance might depend on the interactions between forage source, level, and particle size [35]. Thus, further studies need to focus not only on feeding but also on the forage source and supplementation level.

Rumen ammonia partly comes from the degradation of dietary crude protein available for microbial proteins synthesis [36]. Improved microbial development in the rumen enhances the utilization of NH_3_-N. Hence, to some extent, the concentration of NH_3_-N reflects the development of rumen microbiota [37] and the utilization rate of NH_3_-N [38]. A relatively stable rumen microbiota is gradually achieved as the calves begin to ingest a significant amount of solid feed [39]. Nevertheless, in the early stages of life (three months), the dominant bacteria in the calf rumen are continuously altered [40], especially as an effect of diet changes and age. Feeding high fiber diets increase the abundance of fiber-degrading microbiota [41] and rumen pH resulting in an optimal rumen environment that can stimulate the rapid development of important rumen bacteria [42]. Therefore, providing hay to calves could decrease the concentration and mitigate the negative effects of the rapid accumulation of NH_3_-N in the rumen. Karimizadeh et al. [43] reported higher NH_3_-N concentration when feed block diet compared to pellet or mash diet. Block diet might be devoted to a higher protein intake, which was the same as the starter used in this trial and led to the increased ammonia concentration. 

As calves grow up, the concentration of rumen NH_3_-N decreases gradually. Apart from its utilization by rumen microbiota, NH_3_-N is also absorbed across the rumen wall [44]. Mirzaei et al. [8] reported that rumen corneum thickness decreased in calves fed alfalfa hay with a long particle size (5.04 mm). At the same time the NH_3_-N concentration decreased in the rumen fluid suggesting improved absorption of NH_3_-N across the rumen wall. Our results showed that NH_3_-N was not affected by the length of hay during different periods. Our results imply that OL (10–12 cm) might slightly limit starter intake compared to OS (3–5 cm), thereby reducing energy supply [45].

Compared to hay, the high portion of carbohydrates in calf starter contributes to greater concentrations of VFA during rumen microbial fermentation. Generally, the CON calves had greater concentrations of TVFA compared to hay calves during the whole trial period. This corresponded to low rumen pH, a common feature in calves fed high levels of concentrates. Similar to our TVFA results, Castells et al. [10] reported that calves fed oat hay could lower the retention time of feed (28.4 h for starter vs. 18.8 h for oat hay) in the gastrointestinal tract compared to those fed starter only, thus reduce fermentation time and VFA concentration. Propionate is a key source of energy [45], while butyrate is important in promoting rumen epithelium development [46]. Our study reported no differences in acetate concentrations among treatments during the pre-weaning period and entire trial, while propionate decreased in the calves fed hay during entire trial. Suárez et al. [31] showed that different forage to concentrate ratio affects acetate concentration in the rumen. Hay contains abundant fibers that attract and encourage the growth of cellulolytic bacteria which could produce large amounts of acetate [47] and increase pH. The concentration of acetate increased significantly as well as C2/C3 during post-weaning in the calves fed hay, indicating that the rumen fermentation favored acetate fermentation in these calves. Lower concentrations of butyrate were observed in OL compared to OS calves. The low fiber content in the form of lignin and cellulose [26] or the increased butyrate metabolism in the rumen epithelium [23] in calves fed OS might have contributed to the low butyrate concentrations.

### 3.3. Calf Health

Diarrhea and pneumonia are the most common and important calf diseases on dairy farms throughout the year. Several factors, such as poor passive immunity, milk volume, and environmental conditions, can increase the incidence of diarrhea and pneumonia in dairy calves. Ultimately, sick calves experience a decrease in growth and survival rate. Porter et al. [48] documented that fecal score decreased with increasing dietary fiber (low fiber pellet and low fiber coarse mash vs. high fiber pellet and high fiber coarse mash). In the present study, no differences were found between treatments in diarrhea frequency, which implied that hay did not affect diarrhea. However, higher diarrhea frequency was observed in the pre-weaning period (week 1–8). The results might be partially explained by the lack of active immunity that is yet to be fully established during the peri-weaning period [49]. Although there was no difference in the incidence of pneumonia between groups during pre-weaning, the pneumonia occurrence was higher (CON: 47.92%; OS: 43.36%; OL: 45.13%) compared with other study [50] (pneumonia occurrence: 20.71% before weaning). This may be caused by lower environmental temperature during the trial period (average temperature in October, November, and December and January was 14.5 °C, 7 °C, 0 °C and −1 °C, respectively). Environmental temperature changes are important factors leading to the higher occurrence of calf pneumonia, previous researchers [51,52] also documented higher pneumonia occurrence in the autumn (October–December) compared to spring (April–June).

### 3.4. Calf Behavior

Behavioral responses are the normal animal feedback to the nervous system stimuli, which is important for survival at a particular time or in a certain environment [53]. Our study focused on standing, lying, eating, walking chewing and ruminating, abnormal behaviors, self-grooming, and heading out of the pen. Resting is an important calf behavior that has been associated with improved calf welfare. Previous studies [54] show that increased walking, standing, or starter intake time may enhance the maintenance energy expenditure and heat increment thus reducing feed efficiency. Consequently, calves tend to lie down instead of standing in order to reduce energy consumption. Calves fed with hay spent less time on standing, and more time on lying. Since hay digestion is difficult for young calves [7,11], increased lying time enables the calf to spend more time ruminating [17] as rumination is also an energy-consuming process [55]. Indeed, our study and others have shown that calves fed hay devoted more time to rumination. Interestingly, Terre et al. [33] reported lower-lying time in calves fed hay. The differences in lying and ruminating behavior between the two studies could be linked to how we defined lying time. We considered time spent ruminating while the calf was lying and treated the two activities (chewing and lying) separately [33].

Compared with the CON group, hay fed calves spent less time eating calf starter but their intake was higher. This increase in starter intake could be associated with greater rumen capacity [29]. Consequently, OS and OL calves might require more time than CON to digest the large amounts of calf starter consumed in just a few minutes. However, longer hay supplementation also results in higher rumen fill than shorter hay, which might result in reduced feed intake [56]. It has been shown that calf satiety can reduce the time calves spend performing abnormal behavior (i.e., if the calf licked any surface such as fences, floors, windshields) [57]. Castells et al. [17] also found that calves fed hay devoted less time on abnormal behavior. More self-grooming behavior and less head out of pen were found in both groups of calves fed hay. Self-grooming occurs mainly when the calf is in a frustration mood, while head out of pen reflects the curiosity and distress caused by its separation from other calves [58]. The present study found that providing hay could reduce the time spent on head out of pen which suggests that providing hay to calves can reduce distress in calves, especially at weaning time. However, the concrete connection between these behaviors requires further study. In our study, starter and oat hay were fed separately in two different buckets placed side by side, allowing the calves to consume either feed freely. The feeding protocol was intended to reduce stress (calves can eat according to physiological needs, such as meeting nutrient needs and preventing rumen acidosis) [59] and allow the researchers to observe the feeding behavior among calves easily. However, most calves spilled their hay portion on the ground, making it difficult to accurately calculate the forage to concentrate ratio.

## 4. Materials and Methods

### 4.1. Ethical Statement

Animal care and use were approved by the Ethical Committee of China Agricultural University (Yuanmingyuan Road, Haidian District, Beijing, China; Case number: Aw10601202-1-2; Date of approval: 1 June 2021).

### 4.2. Samples Selection and Treatments

This research was conducted at Zhongyuan Animal Husbandry Co. Ltd. (Shijiazhuang, China) from October 2018 to January 2019 (average temperature in October, November, December, and January was 14.5 °C, 7 °C, 0 °C and −1 °C, respectively) and this work is part of a greater project [60]. Two hundred and ten healthy Holstein female calves (initial BW = 35.8 ± 2.6 kg; serum total protein ≥ 5.5 g/dL) were randomly allocated into three groups: calves fed basic diet (calf starter) without hay (CON) or basic diet with oat hay, as either long (OL: 10–12 cm) or short (OS: 3–5 cm) hay cut. 

The experiment lasted for 70 days (from birth to the end of week 10). Calves were fed 6 L of colostrum in two portions (4 L colostrum within 1 h after birth, 2 L colostrum 8 h later). Pasteurized milk (60 °C, 30 min) was provided twice daily at 07:00 and 14:00 in equal amounts as follows: 6 L/day from days 2 to days 7, 8 L/day from days 8 to 42, 6 L/day from days 43 to 49, 4 L/day from days 50 to 56. Weaning was imposed on day 57 and calves remained in their individual hutches (Hutches were designed with a fenced area, the inside dimensions of the hutch were 215 cm long, 220 cm wide and 136 cm high and the outside fence were 160 cm long, 110 cm wide and 120 cm high; the space between two individual hutches was 80 cm). Clean water, calf starter and oat hay were provided ad libitum. Calf starter was fed from day four while the hay was fed from the second week of life. Same batches of calf starter and oat hay were offered throughout the experimental period. Fresh starter and refusals were weighed daily at 8:00 a.m. after morning milk feeding. Due to the limitations of the hay bucket (Capacity: 5 L; fixed on hutches manually) and the lightness of the hay, most of the calves spilled their portions. Hence, we could not accurately determine the daily intake of hay and these data were excluded in the final analysis. The calf hutches were kept clean and dry throughout the trial. The ingredient and chemical composition of calf starter and oat hay are shown in Table 7.

### 4.3. Feed Analysis and Body Measurements

Representative starter and oat hay samples were collected once a week for further determination of dry matter (DM), crude protein (CP), ether extract (EE), crude Ash (Ash), neutral detergent fiber (NDF), and acid detergent fiber (ADF) following the methods of AOAC [61]. The concentration of calcium, phosphorus, sodium chloride, lysine was recorded from the label on the package. The body weight, body height, body length, heart girth, abdominal girth, and circumference of the cannon bone of the leg of calves were measured before morning feeding on days 1, 14, 28, 42, 56, 63, and 70.

### 4.4. Collection and Determination of the Rumen Fluid Samples

Fourteen (14) healthy Holstein female calves were selected randomly from each treatment for rumen fluid sampling on days 14, 28, 56, 63, and 70. Rumen fluid was collected with esophageal tube (2 mm wall thickness, 6 mm internal diameter; Anscitech Co., Ltd., Wuhan, China) 3 h after morning feeding. The first 20 mL of rumen fluid was discarded to reduce the chances of saliva contamination. The rumen fluid was filtered through four layers of cheese cloth, then divided and placed into two 15 mL centrifuge tubes. The pH value of rumen fluid was measured immediately with a pH meter (HORIBA Advanced Techno Co., Ltd., Osaka, Japan). Rumen fluid was then stored at −20 °C for determination of the concentration of VFA and NH_3_-N with gas chromatography [62] and Phenol-sodium hypochlorite colorimetry [63], respectively.

### 4.5. Evaluation of Calf Health Status

Fecal scores were determined before morning and afternoon milk feeding based on a standardized diarrhea scoring system [64]. The reference criteria for the fecal score included a 4 points scale (1–4 points; calves were considered diarrheic when the calf fecal score was >2). Feces was scored as 1 when calves had a firm but not hard feces; 2 when feces did not hold form and piles but spread slightly; 3 when feces spread readily to about 6 mm depth; and 4 when calves feces had a kind of liquid consistency and easily splatters. The rate and frequency of diarrhea were calculated to reflect the degree of diarrhea in the calves. Pneumonia scores were evaluated based on the scoring system presented by Love et al. [65]. Ocular discharge (any discharge, 2 points), nasal discharge (any discharge, 3 points), coughing (induced or spontaneous, 2 points), ear and head carriage (ear droop or head tilt, 5 points), and respiratory quality (abnormal respiration, 2 points) were considered during the pneumonia scoring. The calves were confirmed positive when their total score was ≥4.

### 4.6. Calf Behavior

The same fourteen calves from which rumen fluid was collected were used to observe calf behavior on days 57, 63, and 70 of the experimental period using a video camera (Hikvision Digital Technology Co., LTD. Hangzhou, Zhejiang Province, China). The duration of a given behavior for each calf was recorded using the time sampling method [66,67], which involves recording a portion of the total time the calf performs a given behavior. We recorded the duration for each behavior within the first 20 min of each hour; a total of 24 20-min durations were recorded throughout the day (24 h/day). These durations were then averaged and multiplied by three. The behaviors studied included standing, lying, eating starter and hay, drinking, walking, rumination, abnormal behavior, self-grooming and heading out of the pen (Table 8).

### 4.7. Statistical Analysis

All raw data was processed using EXCEL. Data of BW, body structure, rumen pH, NH_3_-N, and VFA concentration at days 1, 14, 28, 42, 56, 63, and 70 were analyzed separately in two periods. ADG and starter intake data were pooled and analyzed bi-weekly and weekly, respectively, and then analyzed separately by periods (pre-weaning, days 1–56; post-weaning, days 57–70). The data of calf behavior was recorded for each calf by day (day 57, 63, 70). The above data were analyzed by a mixed model (PROC MIXED, version 9.2; SAS Institute, Inc., Cary, NC, USA) with time as a repeated measure. The model included the fixed effects of treatment, time (day or week) and their interactions (treatment × time), and calf as a random effect. The data of BW, structural measurements, ruminal pH, NH_3_-N, VFA proportion, ADG and starter intake for the entire trial (day 1–70 or week 1–10) used the mixed model with fixed effect of treatment, period (pre-weaning, post-weaning), and their interactions (treatment × period) and calf as a random effect. Fecal scores for each calf were recorded daily and used to calculate diarrhea frequency and diarrhea duration. Data on diarrhea frequency and diarrhea duration were analyzed using a GLIMMIX procedure in SAS (version 9.2, SAS Institute Inc., Cary, NC, USA) with fixed effect of treatment, time (day 1–70) and their interactions (treatment × time), and the random effect of calf within treatment [70]. Data on pneumonia occurrence were analyzed using a chi-square test model (PROC FREQ, version 9.2; SAS Institute, Inc., Cary, NC, USA). *p* < 0.05 showed significant differences, *p* < 0.01 showed extremely significant differences, while trends were indicated as 0.05 < *p* ≤ 0.10.

## 5. Conclusions

Our results showed that feeding oat hay to pre-weaning dairy calves can improve growth performance, rumen fermentation, and reduce abnormal behaviors compared with the starter only. Furthermore, the ADG were significantly improved in calves fed oat hay cut at 3–5 cm during pre-weaning and entire trial. We suggested that feeding short oat hay (3–5 cm) might be the best hay size to feed calves on dairy farms that supply forage to their calves. Further studies are recommended to determine other factors and their interactions that may contribute to better performance in calves.

## Figures and Tables

**Table 1:** Effects of different lengths of hay supplementation in Holstein dairy calves on BW, ADG, and calf starter intake in different periods of study ^1^.

Items	Treatment ^2^			SEM	Contrast *p*-Value				
	CON	OS	OL		T	t	T * t	*p* ^4^	T * *p*
Initial BW (kg)	35.43	35.70	36.01	-	0.46	-	-	-	-
BW (kg)									
Pre-weaning	52.76 ^b^	54.87 ^a^	54.69 ^a^	0.53	<0.01	< 0.01	< 0.01	-	-
Post-weaning	75.41 ^b^	83.05 ^a^	81.24 ^a^	0.90	<0.01	< 0.01	0.42	-	-
Entire trial	64.09 ^b^	68.92 ^a^	67.96 ^a^	0.78	<0.01	-	-	<0.01	<0.01
ADG (kg/d)									
Pre-weaning	0.54 ^c^	0.64 ^a^	0.60 ^b^	0.03	< 0.01	<0.01	< 0.01	-	-
Post-weaning	0.92 ^b^	1.04 ^a^	1.01 ^a^	0.04	< 0.01	<0.01	0.17	-	-
Entire trial	0.72 ^c^	0.84 ^a^	0.80 ^b^	0.014	< 0.01	-	-	<0.01	0.87
Calf starter intake ^3^ (g/d)									
Pre-weaning	174.65 ^b^	226.83 ^a^	202.63 ^ab^	11.87	<0.05	<0.01	<0.01	-	-
Post-weaning	1675.97	1883.45	1790.92	62.28	0.10	<0.01	0.17	-	-
Entire trial	925.31 ^b^	1055.14 ^a^	996.78 ^ab^	35.71	<0.05	-	-	<0.01	0.18

^a,b,c^ Means within a row with different superscripts differ. ^1^ Pre-weaning: from birth to week 8 of age; Post-weaning: from week 8 to 10 of age. Entire trial: from birth to week 10. ^2^ CON = control (basic diet without hay); OS = inclusion of short oat hay (3–5 cm); OL = inclusion of long oat hay (10–12 cm). ^3^ The Calf starter was provided by Yuanxing Co. Ltd. (Hohhot, Inner Mongolia Autonomous Region, China), and contained corn, soybean meal, cotton meal, barley, stone powder, sodium chloride, vitamins and retinoids. Daily individual feed intake (g) = amount of fresh feeds given (g)—amount of feeds refusals (g). ^4^ Data were analyzed for the entire trial (pre-weaning, post-weaning) period. * The interaction between treat and time (T * t) or treat and period (T * *p*).

**Table 2:** Effects of different length of hay supplementation in Holstein dairy calves on body structural growth in different periods of study ^1^.

	Treatment ^2^			SEM	Contrast *p*-Value				
Items	CON	OS	OL		T	t	T * t	*p* ^3^	T * *p*
Body height (cm)									
Pre-weaning	80.36	80.20	80.45	0.30	0.72	<0.01	0.19	-	-
Post-weaning	88.08	88.49	88.42	0.31	0.66	<0.01	0.53	-	-
Entire trial	84.22	84.34	84.43	0.30	0.84	-	-	<0.01	0.23
Body length (cm)									
Pre-weaning	75.04	75.19	75.46	0.22	0.37	<0.01	0.09	-	-
Post-weaning	84.24 ^b^	85.94 ^a^	86.00 ^a^	0.30	<0.01	<0.01	0.84	-	--
Entire trial	79.64 ^b^	80.56 ^a^	80.73 ^a^	0.22	<0.05	-	-	<0.01	<0.01
Heart girth (cm)									
Pre-weaning	86.49	86.97	87.05	0.32	0.35	<0.01	<0.05	-	-
Post-weaning	100.39 ^b^	101.92 ^a^	101.53 ^a^	0.39	<0.05	<0.01	0.97	-	-
Entire trial	93.44 ^b^	94.43 ^a^	94.29 ^a^	0.27	0.08	-	-	<0.01	<0.05
Abdominal Girth (cm)									
Pre-weaning	92.48 ^b^	94.21 ^a^	94.20 ^a^	0.38	<0.01	<0.01	0.07	-	-
Post-weaning	114.35 ^b^	118.50 ^a^	118.14 ^a^	0.60	<0.01	<0.01	0.53	-	-
Entire trial	103.42 ^b^	106.36 ^a^	106.17 ^a^	0.44	<0.01	-	-	<0.01	<0.01
Circumference of cannon bone (cm)									
Pre-weaning	10.74	10.80	10.81	0.04	0.39	<0.01	0.24	-	-
Post-weaning	11.47	11.60	11.59	0.05	0.20	<0.01	0.90	-	-
Entire trial	11.10	11.20	11.20	0.04	0.23	-	-	<0.01	0.40

^a,b^ Means within a row with different superscripts differ.^1^ Pre-weaning: from birth to week 8 of age; Post-weaning: from week 8–10 of age. Entire trial: from birth to week 10. ^2^ CON, control (basic diet without hay); OS, inclusion of short oat hay (3–5 cm); OL, inclusion of long oat hay (10–12 cm). ^3^ Data were analyzed for the entire trial (pre-weaning, post-weaning) period. * The interaction between treat and time (T * t) or treat and period (T * *p*).

**Table 3:** Effects of different lengths of hay supplementation on calf’s rumen pH and NH_3_-N at different stages ^1^.

	Treatment ^2^			SEM	Contrast *p*-Value				
Items	CON	OS	OL		T	t	T * t	*p* ^3^	T * *p*
pH									
Pre-weaning	6.26 ^b^	6.43 ^a^	6.44 ^a^	0.05	<0.05	<0.01	0.69	-	-
Post-weaning	6.13 ^b^	6.41 ^a^	6.32 ^a^	0.05	<0.01	0.72	0.46	-	-
Entire trial	6.19 ^b^	6.42 ^a^	6.38 ^a^	0.03	<0.01	-	-	<0.05	0.44
NH_3_-N (mmol/L)									
Pre-weaning	17.54 ^a^	13.03 ^b^	13.63 ^b^	0.59	<0.01	<0.01	<0.01	-	-
Post-weaning	9.02 ^a^	6.23 ^b^	5.64 ^b^	0.37	<0.01	<0.01	0.36	-	-
Entire trial	13.29 ^a^	9.65 ^b^	9.64 ^b^	0.52	<0.01	-	-	<0.01	0.49

^a,b^ Means within a row with different superscripts differ. ^1^ Pre-weaning: from birth to week 8 of age; Post-weaning: from week 8 to 10 of age. Entire trial: from birth to week 10. ^2^ CON, control (basic diet without hay); OS, inclusion of short oat hay (3–5 cm); OL, inclusion of long oat hay (10–12 cm). ^3^ Data were analyzed for the entire trial (pre-weaning, post-weaning) period. * The interaction between treat and time (T * t) or treat and period (T * *p*).

**Table 4:** Effect of different lengths of hay supplementation on calf’s rumen volatile fatty acids at different stages ^1^.

	Treatment ^2^			SEM	Contrast *p*-Value				
Items	CON	OS	OL		T	t	T * t	*p* ^3^	T * *p*
Acetate (%)									
Pre-weaning	50.61	51.39	51.71	0.009	0.81	<0.01	0.28	-	-
Post-weaning	44.44 ^b^	49.05 ^a^	49.11 ^a^	0.009	<0.05	<0.01	0.67	-	-
Entire trial	47.50	50.41	50.61	0.014	0.24	-	-	<0.05	0.67
Propionate (%)									
Pre-weaning	26.39	26.23	28.40	0.007	0.34	<0.01	0.07	-	-
Post-weaning	41.29 ^a^	34.67 ^b^	37.10 ^b^	0.009	<0.01	0.61	0.20	-	-
Entire trial	33.86 ^a^	30.46 ^b^	32.71 ^a^	0.009	<0.05	-	-	<0.01	<0.05
Butyrate (%)									
Pre-weaning	12.31 ^a^	13.30 ^a^	11.34 ^b^	0.020	<0.05	<0.01	0.06	-	-
Post-weaning	10.42 ^b^	13.39 ^a^	11.04 ^b^	0.007	<0.05	<0.05	0.23	-	-
Entire trial	12.30	13.57	11.55	0.007	0.12	-	-	0.34	0.24
Valerate (%)									
Pre-weaning	9.38	9.05	8.34	0.007	0.50	<0.01	0.38	-	-
Post-weaning	3.85	2.89	2.75	0.004	0.16	<0.01	0.77	-	-
Entire trial	6.68	5.95	5.53	0.005	0.29	-	-	<0.01	0.91
TVFA (mmol/L)									
Pre-weaning	87.56 ^a^	82.69 ^ab^	73.92 ^b^	3.79	<0.05	<0.01	0.65	-	-
Post-weaning	189.03	172.66	170.19	8.58	0.33	<0.05	0.87	-	-
Entire trial	133.62 ^a^	123.66 ^ab^	119.99 ^b^	4.86	0.12	-	-	<0.01	0.85
C2/C3									
Pre-weaning	2.55	2.43	2.19	0.23	0.30	<0.01	0.15	-	-
Post-weaning	1.12 ^b^	1.49 ^a^	1.42 ^a^	0.09	<0.05	0.32	0.61	-	-
Entire trial	1.82	1.96	1.81	0.13	0.67	-	-	<0.01	0.24

^a,b^ Means within a row with different superscripts differ.^1^ Pre-weaning: from birth to week 8 of age; Post-weaning: from week 8 to 10 of age. Entire trial: from birth to week 10. ^2^ CON, control (basic diet without hay); OS, inclusion of short oat hay (3–5 cm); OL, inclusion of long oat hay (10–12 cm). ^3^ Data were analyzed for the entire trial (pre-weaning, post-weaning) period. * The interaction between treat and time (T * t) or treat and period (T * *p*).

**Table 5:** Effect of different lengths of hay supplementation on calf’s diarrheal frequency, diarrheal duration and pneumonia occurrence during different periods ^1^.

	Treatment ^2^			SEM	Contrast *p*-Value		
Items	CON	OS	OL		T	*p* ^5^	T * *p*
Diarrhea Frequency (%) ^3^							
Pre-weaning	7.04%	7.29%	6.91%	0.006	0.89	-	-
Post-weaning	2.86%	2.15%	2.64%	0.005	0.76	-	-
Entire trial	4.95%	4.72%	4.78%	0.006	0.95	<0.01	0.70
Diarrhea Duration (days) ^4^							
Pre-weaning	3.94	4.08	3.87	0.33	0.89	-	-
Post-weaning	0.40	0.30	0.37	0.09	0.77	-	-
Entire trial	4.34	4.38	4.24	0.35	0.95	-	-
Pneumonia Occurrence (%)							
Pre-weaning	47.92	43.36	45.13	-	0.49	-	-

^1^ Pre-weaning: from birth to week 8 of age; Post-weaning: from week 8 to 10 of age. Entire trial: from birth to week 10. ^2^ CON, control (basic diet without hay); OS, inclusion of short oat hay (3–5 cm); OL, inclusion of long oat hay (10–12 cm). ^3^ Diarrhea Frequency, ∑Incidence of diarrhea in experimental calves/(Calf numbers × days of trial) × 100%. ^4^ Diarrhea Duration, ∑Number of days with diarrhea in each calf/Calf numbers × 100%, there is only one period for this indicator, since no calves have pneumonia during post-weaning. ^5^ Data were analyzed for the entire trial (pre-weaning, post-weaning) period. * The interaction between treat and period (T * *p*).

**Table 6:** Effects of different lengths of hay supplementation on calf’s behavior.

	Treatment ^1^			SEM	Contrast *p*-Value		
Items	CON	OS	OL		T	t	T * t
Standing (min/d)	461.60 ^a^	426.39 ^b^	430.33 ^b^	16.47	<0.05	0.27	0.81
Lying (min/d)	877.09 ^b^	931.03 ^a^	910.06 ^b^	35.65	<0.05	0.65	0.15
Eating starter (min/d)	123.38 ^a^	99.418 ^b^	101.86 ^b^	6.45	<0.05	0.77	0.87
Eating Hay (min/d)	-	100.82	94.06	8.45	-	0.58	0.35
Drinking (min/d)	16.73	14.79	18.36	3.26	0.46	0.22	0.55
Walking (min/d)	20.65	15.05	15.63	2.02	0.16	0.82	0.22
Chewing and Ruminating (min/d)	133.20 ^b^	291.41 ^a^	263.05 ^a^	15.77	<0.01	0.17	0.49
Abnormal Behavior ^2^ (min/d)	206.72 ^a^	78.87 ^b^	72.65 ^b^	9.36	<0.01	0.60	0.09
Self-Grooming ^3^ (min/d)	12.92	19.43	15.88	2.25	0.16	0.23	0.35
Head out of Pen (min/d)	180.38 ^a^	127.91 ^b^	132.45 ^b^	8.10	<0.01	0.66	0.89

^a,b^ Means within a row with different superscripts differ. ^1^ CON, control (basis diet without hay); OS, inclusion of short oat hay (3–5 cm); O, inclusion of long oat hay (10–12 cm). ^2^ Abnormal behavior mainly for non-nutritive oral behavior. ^3^ Self-grooming: calf licked itself with its tongue. * The interaction between treat and time (T * t).

**Table 7:** Ingredient and chemical composition (as % of DM) of calf starter and oat hay.

Component ^1^	Stater ^2^	Oat Hay ^3^
DM (%)	89.48	93.20
CP (%, DM)	29.86	6.18
EE (%, DM)	2.27	2.75
Ash (%, DM)	7.49	4.48
NDF (%, DM)	9.50	44.14
ADF (%, DM)	8.47	33.70
ME (Mcal/kg) ^4^	3.23	2.87
Calcium (%, DM)	0.3–2.0	-
Phosphorus (%, DM)	≥0.2	-
Sodium chloride (%, DM)	0.2–2.4	-
Lysine (%, DM)	≥0.2	-

^1^ Regular nutrients measured included dry matter (DM), crude protein (CP), ether extract (EE), ash, neutral detergent fiber (NDF), and acid detergent fiber (ADF). Concentration of calcium, phosphorus, sodium chloride, lysine was recorded from the label on the package. ^2^ Calf starter (Yuanxing Co. Ltd., Hohhot, Inner Mongolia Autonomous Region, China) contained corn, soybean meal, cotton meal, barley, stone powder, sodium chloride, vitamins and retinoids, enzyme preparation and antioxidants.^3^ Oat hay was cut into two lengths (3–5 cm; 10–12 cm) using a stationary mixer (20 m^3^, Trioliet Co. Ltd., Oldenzaal, The Netherlands) at different powers. ^4^ Calculated metabolic energy. DE (Mcal/kg) in feeds was calculated by NRC Nutrient Requirements of Dairy Cattle v. 1.1.9; ME(Mcal/kg) = 1.01 × DE (Mcal/kg) − 0.45 [14].

**Table 8:** Definitions of the examined behaviors.

Behavior	Definition of the Behavior
Standing ^1^	Four hooves on the ground, whether moving or not
Lying ^1^	Lying on the sternum with head held in a raised position or down
Eating starter ^1^	Head in starter feed bucket accompanied by chewing movements
Eating Hay ^1^	Head in hay feed bucket accompanied by chewing movements
Drinking ^1^	Mouth around drinker
Walking ^1^	Stepping and moving
Chewing and Ruminating ^1^	Chewing irregularly and repeatedly without food in the mouth
Abnormal Behavior ^2^	Calf licked any surface such as fences, floors, windshields
Self-Grooming ^1^	Calf licked itself with its tongue
Head out of Pen ^3^	Calf’s head out of the pen to look around and do not engage in any feeding activities

^1^ Adapted from [68]. ^2^ Adapted from [17]. ^3^ Adapted from [69].

## Data Availability

All the data are already provided in the main manuscript. Contact the corresponding author if further explanation is required.
